# Langmuir films at the oil/water interface revisited

**DOI:** 10.1038/s41598-019-38674-9

**Published:** 2019-02-19

**Authors:** Milagro Mottola, Benjamín Caruso, Maria A. Perillo

**Affiliations:** 1 Universidad Nacional de Córdoba. Facultad de Ciencias Exactas, Físicas y Naturales. Depto. de Química, Cátedra de Química Biológica. Córdoba, Argentina. Av. Vélez Sarsfield 1611, X5016GCA Córdoba, Argentina; 2 CONICET, Instituto de Investigaciones Biológicas y Tecnológicas (IIBYT). Córdoba, Argentina. Av. Vélez Sarsfield 1611, 5016 Córdoba, Argentina

## Abstract

We studied monomolecular layers at the oil/water interface (O/W_int_) in a Langmuir interfacial trough using egg-yolk phosphatidylcholine (EPC) (the model phospholipid) and Vaseline (VAS) as oil phase. The temporal dynamics in the surface pressure (π) evolution depended on the method (spreading/adsorption) used for monolayers preparation and reflected the different distribution of EPC between all the system compartments (bulk phases and interfaces). We distinguished between EPC located either stable at the interface or hopping between the interface and bulk phases. The size order of the apparent mean molecular area, at constant π, of EPC at different interfaces (EPC_O/W_ > EPC/VAS_0.02;A/W_ > EPC_A/W_), suggested that VAS molecules intercalated between the hydrocarbon chains of EPC_O/W_, at a molar fraction *x*_VAS_ > 0.02. However, EPC/VAS_0.02;A/W_ showed the highest compressional free energy. This leaded us to study the EPC/VAS_0.02_ mixture at A/W by Brewster Angle Microscopy (BAM), finding that upon compression VAS segregated over the monolayer, forming non-coalescent lenses (as predicted by the spreading coefficient S = −13 mN/m) that remained after decompression and whose height changed (increase/decrease) accompanied the compression/decompression cycle. At the O/W_int_, while some VAS molecules remained at the interface up to the collapse, others squeezed out towards the VAS bulk phase with an energy requirement lower than towards the air.

## Introduction

Monolayers at the interface between two immiscible solvents (liquid/liquid interface) are an interesting model system to investigate the structure and stability of nanoemulsions at the molecular level. This knowledge is relevant in many phenomena that are crucial in areas as diverse as life sciences, environmental sciences, and technology. The variety of studies found in literature include not only monolayers of surfactants of different chemical nature (e.g. lipids^1–3^, asphaltenes^4^, proteins^5,6^ and polymers^7^) but also of solid particles (nanoparticles, particles in the colloidal size range and larger)^8,9^. While one of the bulk liquid is usually water the other liquid phase can be volatile solvents (e.g. chloroform, toluene^4^, benzene^10^), alkanes of different chain lengths^2^ and natural triglyceride oils^11^.

Although a liquid interface can be viewed as a region segregating two bulk liquids, sometimes it does not prevent the transfer of mass and energy between them. So, depending on the chemical composition of the surfactants and of the hydrophobic liquid phase, monolayers can behave as insoluble or soluble films^12^.

Despite the immense interest in mixed monolayers at the oil-water interface, very little information on the use of a Langmuir interfacial film balance is available because of the experimental difficulties and challenges not detailed or not revealed in the literature. Most experiments to measure surface pressure vs. molecular area (π − A) isotherms have been performed by the pendant drop technique^13^ and also with rectangular troughs of different designs^4,14,15^. The material of the sensor (Wilhelmy plate) used for π measurements varies among published works from Teflon^TM^ ^14^, platinized platinum^16^, carbon^17^, hydrophobic mica covered with carbon-black from a butane/air flame^15^ or filter paper^4^. The choice of a proper material for the Wilhelmy plate is crucial to satisfy the condition of zero contact angle (*θ* = 0°) between the sensor and the liquids^16,18^. This important technical detail is usually not sufficiently highlighted. The non-compliance of *θ* = 0° may have consequences not always evidenced, mainly when the surfactant/liquids combination is a system not studied before. Moreover, up to our knowledge, regarding monolayers at the oil/water interface (O/W) neither the rationale behind the methods applied to build them up nor the analysis of their stability limits have received enough attention.

In the present paper we made a thorough comparative analysis of different techniques used for the preparation of Langmuir films at the oil-water interface (LF_O/W_) using egg-yolk phosphatidylcholine (EPC) as a model phospholipid and Vaseline (VAS) as the oil phase (EPC_O/W_). In this model, we evaluated the dynamic behavior and stability of EPC_O/W_ and calculated molecular, rheological and thermodynamic parameters from compression isotherms. Furthermore, in LF_O/W_, water molecules from the aqueous phase can be structured at the EPC’s polar head groups and molecules from the oil phase may be inserted between the EPC hydrocarbon chains. Thus, an EPC_O/W_ monolayer would be considered a pseudo-binary mixture of EPC and VAS. So, to understand the behavior of the EPC_O/W_ monolayer, we tried to model it through Langmuir films of EPC/VAS mixtures at the air-water interface (EPC_A/W_).

## Results and Discussion

Firstly, we analyzed different conditions to build up the Langmuir films at the O/W interface.

### Adsorbed monolayers (AM)

**Figure 1:**
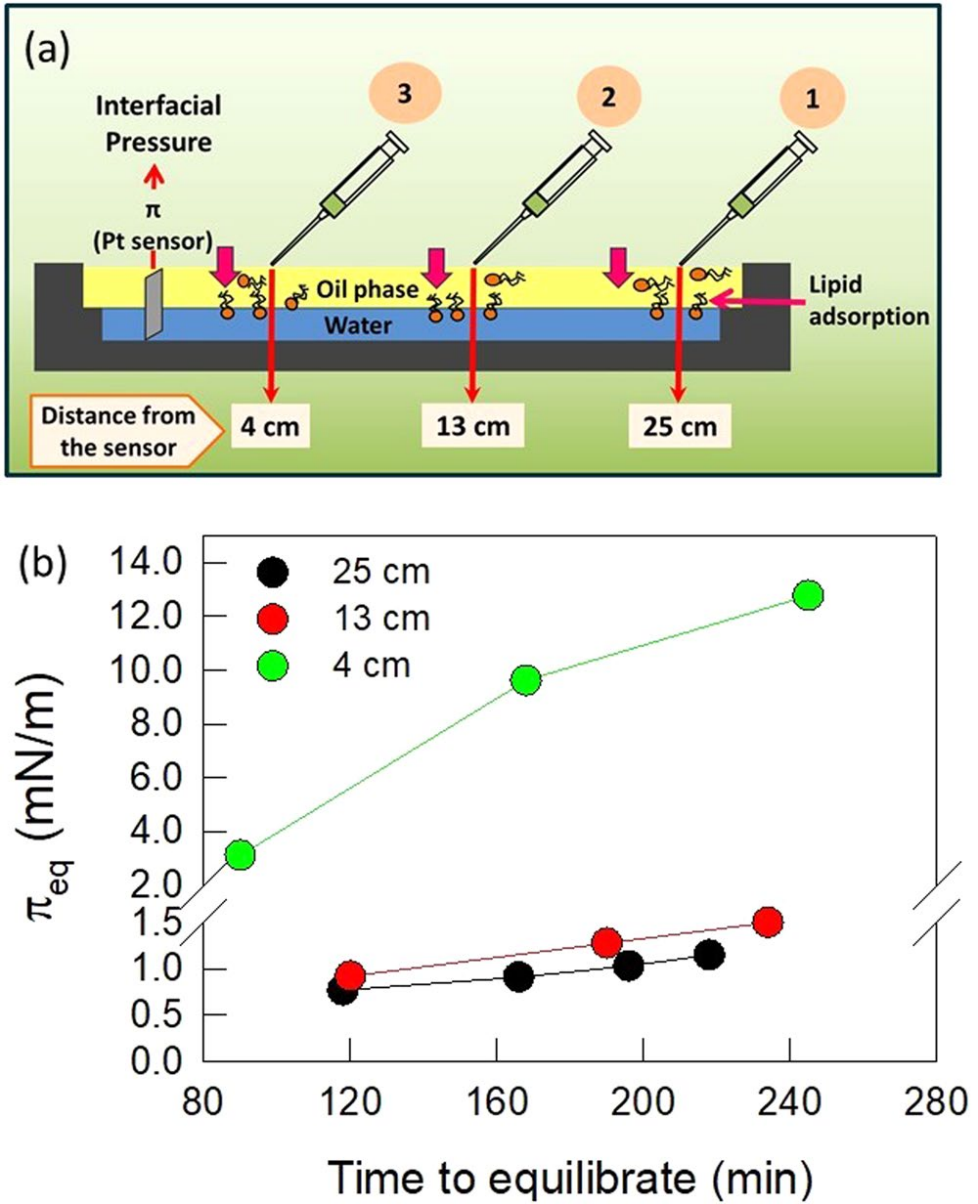
Successive spreads of EPC at the A/O interface, at different distances from Wilhelmy plate. (**a**) Schematic representation of the experimental setup. (**b**) Equilibrium surface pressure at the O/W interface reached after several successive 1 μL-spreads of 10 mM EPC solution, at the A/O interface, as a function of the time length required to reach each equilibrium. Numbers in the graph indicate the distance between the Wilhelmy plate and the point of spreading. Data correspond to representative experiments.

In a first experiment, a fixed volume (1 µL) of 10 mM EPC solution was spread several times at the O/W interface. The equilibrium lateral surface pressure (π_eq_) at the O/W interface vs. the time required to achieve such π value, is depicted in Fig. 1. This was done at different distances from the Wilhelmy plate (Pt sensor) whose position was taken as a reference point (0 cm) (Fig. 1a).

As a general trend, π increased as a function of the number of spreads. The π_eq_ values reached were significantly higher and were achieved in shorter time lengths when the spreading point was closer to the Wilhelmy plate (Fig. 1b). It is noteworthy that the process was very slow, since after the first spread of 1 μL EPC solution it took very long times (90 or 120 min) to reach π_eq_ = 3 or 0.76 mN/m when the spreading was performed at 4 or 25 cm from the sensor, respectively. These results clearly reflected restrictions to EPC diffusion to which contributed 3D diffusion along the upper oil phase and 2D diffusion at both the O/W and air-oil (A/O) interfaces.

**Figure 2:**
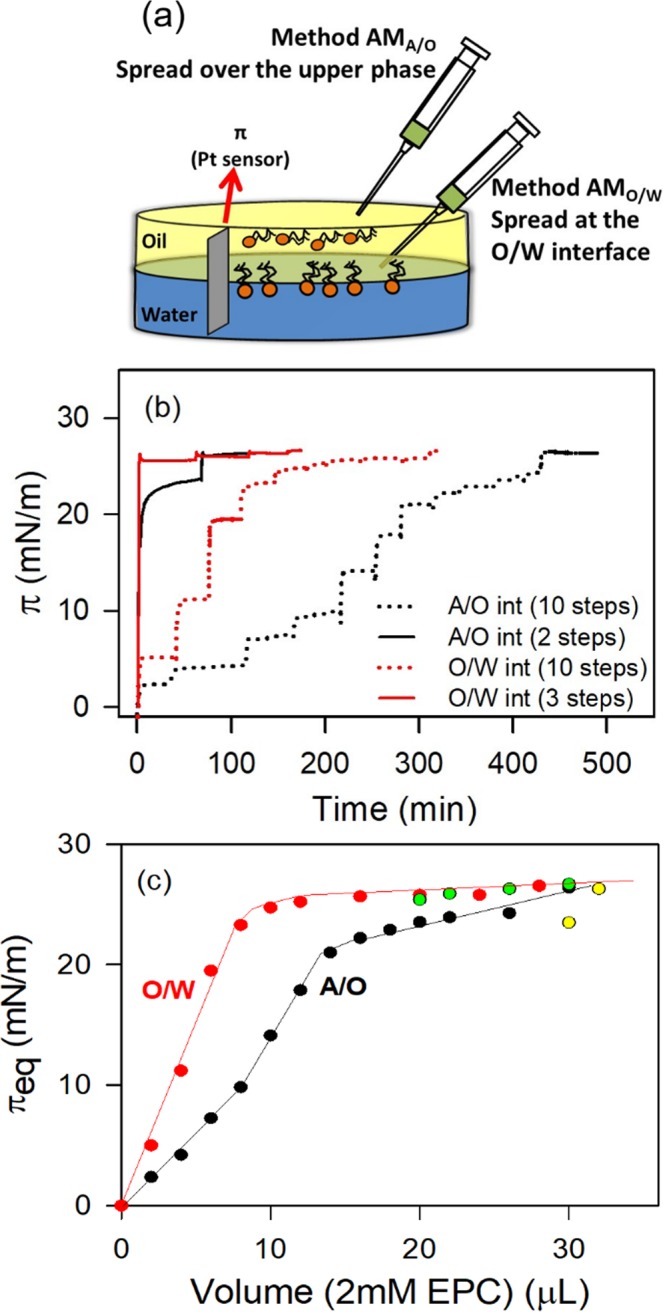
EPC adsorption at the VAS/W interface. (**a**) Schematic representation of the experimental setup. Upper phase: volume 12 ml; total area: 21.23 cm^2^. (**b**) Interfacial Pressure vs Time plots, spread by method AM_A/O_ (— and **....**) (at the A/O interface) or by method AM_O/W_ ( and ) (at the O/W interface) in 2 (full lines) or in several steps (dotted lines). Curves are representative experiments, which vary by less than 2 mN/m from their corresponding replicas. T = 25 ± 1 °C. Yellow and green points correspond to experiments with 2–4 spread steps in O/W and A/W interfaces, respectively.

To compare the performance of methods AM_A/O_ and AM_O/W_, adsorption kinetics studies were performed (Fig. 2a). For this experiment, the EPC solution was spread (in 2, 3 or 10 steps) over the upper phase (Method AM_A/O_) or into the previously formed O/W interface (Method AM_O/W_) until reaching the equilibrium pressure (π_eq_ ~ 26.4 ± 0.1 mN/m). If the addition of EPC solution was done in 2–3 steps (Fig. 2b, solid lines), there was a difference of 4 μL between both adsorption methods to reach the same π_sp_. While the AM_A/O_ method required **34** **μL** of 2 mM EPC to reach the π_sp_ (one spread of 30 μL to reach 23.5 mN/m plus another 2 × 2 μL-spreads to complete the process), the AM_O/W_ method required **30** **μL** (20 μL to reach 25.6 mN/m plus 2 μL plus 2 × 4 μL-spreads). This suggested that with AM_A/O_ a fraction of the lipid molecules spread at the A/VAS interface could not reach the VAS/W interface thus requiring more molecules than with method AM_O/W_ to obtain the π_sp_. Since π-MMA compression isotherms indicated that lipid molecules are not retained at the A/VAS interface (see isotherms at the A/W interface) it follows that the preparation of EPC_O/W_ through the AM_A/O_ method implies that a percentage of molecules remain dissolved in the bulk of the upper phase.

A further analysis of the experiments performed through the addition of EPC in 10 steps (Fig. 2c) allowed demonstrating that, compared with method AM_A/O_, by the method AM_O/W_ π grew up more steeply. Hence, the plateau in the π vs. Volume plot in the case of AM_O/W_ method was reached with a lower volume of the 2 mM-EPC solution (12 μL) than with AM_A/O_ method (28 μL). However, the 12 μL- aliquot of 2 mM-EPC contained 1.44 × 10^16^ EPC molecules which, at π_sp_ (MMA = 0.93 nm^2^/molec, taken from isotherms at the O/W interface), would be able to form a monolayer with a total area (134 cm^2^) which is significantly bigger than the available area of the Petri dish used in these experiments (total area = 21 cm^2^). In other words, in order to achieve a monomolecular layer at π_sp_, these amount of molecules should be packed at a surface molecular density (15.8 Å^2^/molec.) which is significantly higher than what would be expected from the π-Mma isotherm of EPC_O/W_ so, they either may not be arranged in a monomolecular layer or may be partially partitioned towards one or both of the bulk phases. Although this set up did not allow a thorough control of molecular deposition, it provided useful semi-quantitative information of the differential behavior of AM_A/O_ and AM_O/W_ techniques.

**Figure 3:**
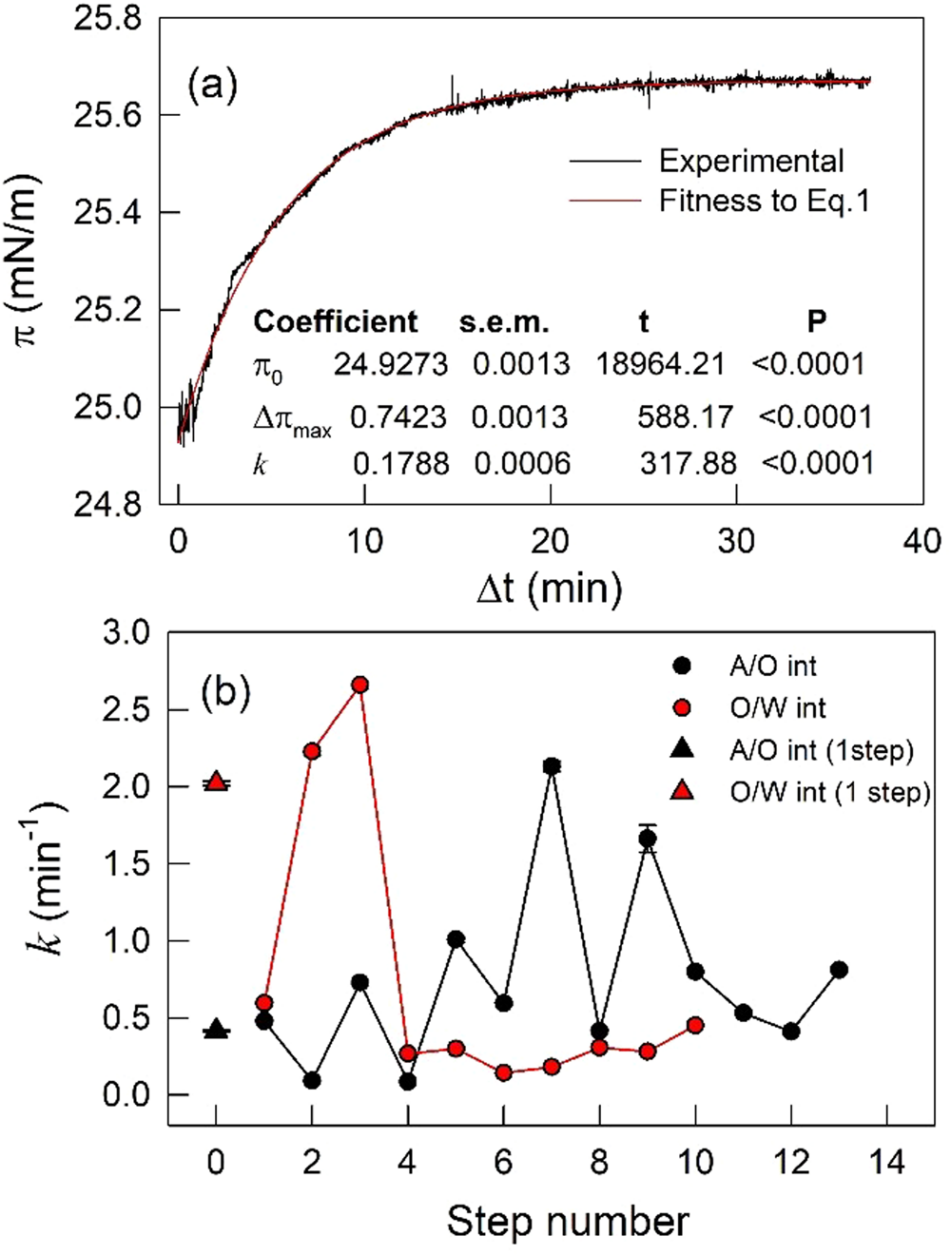
Kinetics of EPC monolayer formation at the VAS/W interface. Representative fit (), by the least squares method, of Eq. 1 to raw data (—) corresponding to each one of the spreading steps shown in (**b**) the statistical significance of the parameters is also included. (**b**) Time constant *k* determined at each spreading step as indicated in (**a**). Error bars are within the symbols’ size.

Statistically significant fits of each step of the spreading shown in Fig. 2b, could be achieved using Eq. 1 (Fig. 3a) and the spreading kinetics could be characterized by the time constant *k* (Fig. 3b).

The time constant for reaching π_sp_ in one step by the adsorption at the A/O interface (AM_A/O_) (*k* = 0.48 min^−1^) was near 4 times lower than *k* = 2 min^−1^ for preparing monolayers by the adsorption at the O/W interface (AM_O/W_). This can be explained by the fact that the former includes a diffusion step of EPC along the upper phase which is absent in the latter.

When the monolayer preparation occurred in several steps through AM_O/W_ method, the adsorption at the O/W interface, initially, exhibited an increasing *k* values, up to *k* = 2.7 min^−1^, letting achieve a 70% of π_sp_. After that, the adsorption rates of the following EPC aliquots added slowed down significantly with k values ~0.3 min^−1^ and remained constant up to the π_sp_. This reflected the higher activation energy (lower 2D diffusion coefficient) required by new added molecules to enter an occupied but non-saturated interface. Also, it suggests the occurrence of a cooperative phenomenon in the initial stages of adsorption, where the first adsorbed molecules would be lowering the lag time required for the detection of subsequent molecules adsorption. Conversely, the adsorption at the A/O interface (AM_A/O_) in several steps exhibited oscillating *k* values along the addition of successive EPC aliquots suggesting an emerging nonlinear global response of the combined 2D plus 3D diffusion phenomena which would deserve further analysis (beyond the scope of the present work).

The 2D-diffusion between two liquid phases can be defined from the Einstein’s equation with *D* = *k*_B_*T*/*λ* with a modified hydrodynamic drag term *λ* = 8(*η*_1_ + *η*_2_)*R* which depends on the viscosity of each liquid (*η*_1_ and *η*_2_) and the radius (*R*) of the diffusing molecule (see Supplementary Information for further information) and dominates at low interfacial viscosities. At the interface between water and a high viscous solvent it was suggested that molecules exhibited a sort of “hopping” mode mechanism of interfacial diffusion that involved transition states where the adsorbate molecule became transiently detached from the surface^19^. This would be consistent with a non-monolayer organization of EPC at the O/W interface suggested above and with the irregular rate constants *k* of EPC adsorption through AM_A/O_ method.

Hence, we discarded method AM_A/O_ because of its impossibility to assure a quantitative molecular spreading and the complexity in its spreading kinetics which would difficult the attainment of molecular parameters such as mean molecular area.

### Spread (SM) and adsorbed (AM_O/W_) monolayers at the O/W interface

**Figure 4:**
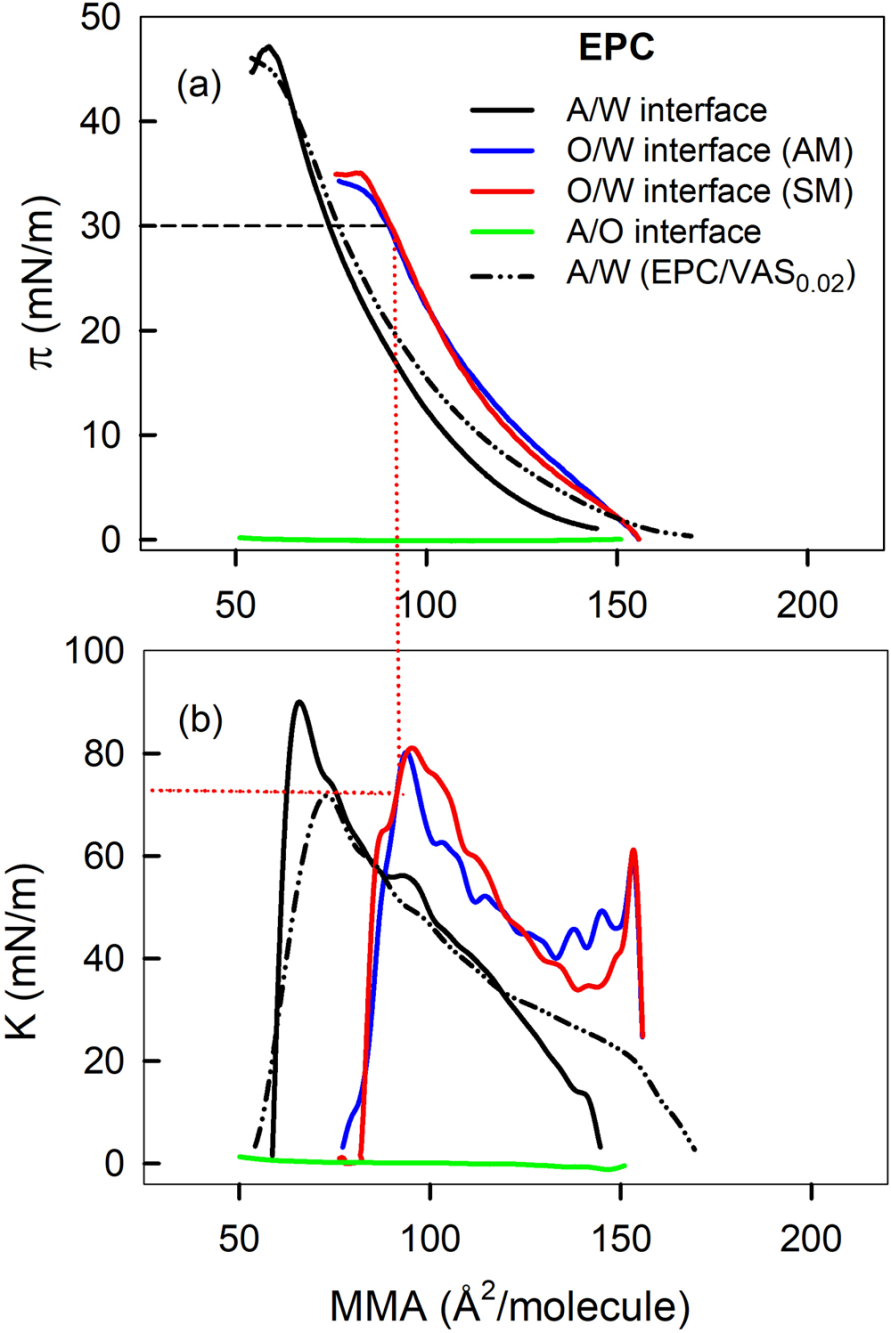
Surface pressure (**a**) and Compressibility modulus (**b**) vs. mean molecular area. Monolayers of EPC, pseudo binary mixture of EPC and VAS at 0.02 molar fraction, were prepared at the air/water (A/W) and/or at the Vaseline/water (O/W) interface. Straight lines illustrate the procedure to obtain K values at 30 mN/m from successive interpolations in π-MMA isotherms and K-MMA, respectively, obtained in each experimental condition.

When using the Teflon trough with an area 10 times bigger than the previously shown set up, satisfactory monolayers could be assembled with both AM_O/W_ and SM methods when the amount of molecules spread leads to an initial MMA higher than the lift-off area. The technique based on the assembly of monolayers by the SM method (spreading at the A/W interface before the addition of the upper oil phase) requires considerable care to prevent the disturbance of the film and the migration of molecules away from the O/W interface. Taking this issue into account, a comparative analysis of AM_O/W_ and SM techniques were performed through surface pressure vs. mean molecular area isotherms (π-MMA) (Fig. 4a). Since the monolayer at the O/W interface includes not only EPC but also molecules from the upper phase, in this case the calculated MMA of EPC should be considered as an apparent MMA.

It was shown that EPC did not form stable monolayers at the A/O interface as evidenced by the fact that, upon compression, the film π remains constant near zero (Fig. 4a, green line) indicating that molecules spread at the interface are being squeezed out. This implies that EPC does not partition to the A/O interface and indicates that, when monolayers are prepared through the AM_A/W_ technique, the whole population of EPC molecules distributes between the liquid oil upper phase and the monolayer at the liquid-liquid interface. π-MMA compression isotherms of films at the O/W interface prepared by AM_O/W_ and SM technique were superimposable (Fig. 4a, blue and red lines) demonstrating that both methods for monolayer preparation at the O/W interface had a similar spreading efficiency. So, as the later method was easiest to perform, it was selected for all the subsequent compression experiments.

Monolayers at the O/W interface exhibited π-MMA compression isotherms more expanded and with lower collapse pressure (π_c_) than isotherms of monolayers prepared at the A/W interface. The latter might be interpreted as a lower stability of the film at the O/W than that at the A/W interface, if the usual rationale is applied. However, the upper limit of π that any film can achieve at a certain interface is the surface tension of such clean interface (γ_0_ in Eq. 3). For the A/W and the VAS/W interfaces, γ_0_ values are γ_0,A/W_ = 72 and γ_0,VAS/W_ = 37 mN/m and the EPC collapse pressures were π_c,A/W_ = 61 and π_c,VAS/W_ = 34 mN/m. Then, π_c,A/W_ and π_c,VAS/W_ would be representing ~85% and ~92% of their respective γ_0_, (see Fig. S1, Supplementary Information), implying that EPC at the O/W would not be less stable than at the A/W interface

The compressibility modulus K, calculated from Eq. 4, is depicted as a function of MMA in Fig. 4b. This parameter reflects qualitatively the physical state of the monolayer and can help identify the occurrence of bidimensional phase transitions. High K value (low compressibility) means that molecules are tightly packed and that the cohesive forces are considerable. K is usually employed to categorize the state of Langmuir monolayers. For monolayers at the air-water interface, a K value of the same magnitude as the π is associated with a gaseous state (G), while for liquid-expanded (LE) films K ranges from 12.5 to 50 mN*/*m and for liquid-condensed (LC) state is characterized by higher K values (within 100–250 mN/m), according to Davies and Rideal’s^20^. Assuming that this rule applies for films at the O/W interface, it can be concluded that all the samples studied changed from G to LE phase along the whole isotherm.

All the films analyzed followed a smooth increase in K along compression up to the onset of the collapse. In some of the isotherms obtained with the EPC/VAS_0.02_ mixture at A/W interface we seldom detected an irregularity between 9 and 12 mN/m (not shown) that resembled a transition that might be associated with the start of the exclusion of the excess of VAS from the monolayer towards a liquid VAS phase. This VAS excess should grow upon compression and remain in equilibrium with an EPC/VAS_A/W_ monolayer of constant composition. This hypothesis is suggested from the behavior of triglyceride/phospholipid films as reflected by their π-composition phase diagrams (Caruso *et al*.^21^ and refs therein) and was confirmed through microscopy studies (see below).

A synthesis of molecular, thermodynamic and rheological data, at 20 and 30 mN/m, of all the monolayers evaluated in Fig. 4 is shown in Table 1. In general terms, compared with monolayers at the A/W, EPC monolayers at the O/W at 20 mN/m were more expanded (higher MMA) and had a similar compressional modulus (K). At 30 mN/m the difference in MMA was reduced while K remained similar for both monolayers. Interestingly, the mixture EPC/VAS_0.02;A/W_ exhibited the lowest K (highest compressibility) at both π.

**Table 1:** Thermodynamic and rheological analysis of Langmuir films.

System composition	MMA (Å^2^/molec)	K^a|^ (mN/m)	MMA (Å^2^/molec)	K^a|^ (mN/m)	Phase state	ΔG_comp_^b^ (cal/mol)
	20 mN/m		30 mN/m			
EPC_A/W_	87 ± 1	61 ± 3	74 ± 1	75 ± 4	LE	410 ± 10
EPC/VAS_0.02 A/W_	91 ± 1	45 ± 3	75 ± 2	58 ± 3	LE	527 ± 5
EPC_O/W_ (AM)	104 ± 1	62 ± 1	90 ± 1	71 ± 3	LE	465 ± 5
EPC_O/W_ (SM)	104 ± 1	68 ± 5	91 ± 1	73 ± 1	LE	451 ± 9

Data were taken from compression isotherms of monolayers of EPC or a pseudo binary mixture of EPC and VAS at 0.02 molar fraction, prepared at the air/water (A/W) or at the VAS/water (O/W) interface. Values are the mean ± S.E.M. of at least two independent experiments. T = 25 ± 1 °C. ^a^Calculated from Eq. 4. ^b^Calculated from Eq. 5 by integrating compression isotherms between 2 and 20 mN/m. The molar fraction of VAS in the EPC:VAS mixture, is indicated as subindex. Phases VAS, W and A, correspond to Vaseline, water and air, respectively.

The *ΔG*_*comp*_ of a film is a complex quantity that reflects not only the energy balance between intermolecular interactions and the loss of entropy upon compression but also the energy related to transitions (not limited to bidimensional phase transitions). The *ΔG*_*comp*_ calculated for EPC monolayers at O/W is higher than at A/W interfaces. Assuming that this monolayer includes molecules from the upper phase, its *ΔG*_*comp*_ possibly contains an energy component required for the squeezing out of VAS molecules from the monolayer to a liquid alkane phase. In turn, the *ΔG*_*comp*_ for the EPC/VAS_0.02,A/W_ monolayer is the highest among all the monolayers studied. It would contain an energy component associated to the transition identified through the K-MMA isotherm, also due to the squeezing out of VAS molecules from the monolayer. Comparing EPC_O/W_ and EPC/VAS_0.02,A/W_, it results intriguing that being the latter the most compressible, at the same time it requires more energy to suffer the same compression (between 2 and 20 mN/m). If in both cases the process of compression implies the exclusion of VAS molecules from the monolayer it seems that the presence of the liquid alkane phase lying over the monolayers pays a facilitating effect. This is further analyzed in the following sections.

The stability of the monolayers was also evaluated through compression-decompression (C-D) cycles. The EPC monolayers at the O/W interface exhibited significant hysteresis, usually positive and sometimes negative, depending on the material of the Wilhelmy plate (paper, Pt, platinized Pt), the compression rate and the π reached before starting the decompression. Reproducible conditions were reached with platinized Pt.

### EPC/VAS Langmuir films at the air/water interface: an experimental model of EPC monolayers at the oil-water interface

Assuming a tentative composition of the EPC monolayer at the oil/water interface (EPC_O/W_), we formulated an EPC mixture with Vaseline at a molar fraction *x*_vas_ = 0.02 (EPC/VAS_0.02,A/W_) and studied the monolayer formed at the air/water interface. The compression isotherm of this mixture is shown in Fig. 4a (broken black line). At low π, the EPC/VAS_0.02,A/W_ isotherm was more expanded than pure EPC at A/W suggesting the incorporation of VAS molecules into the monolayer.

**Figure 5:**
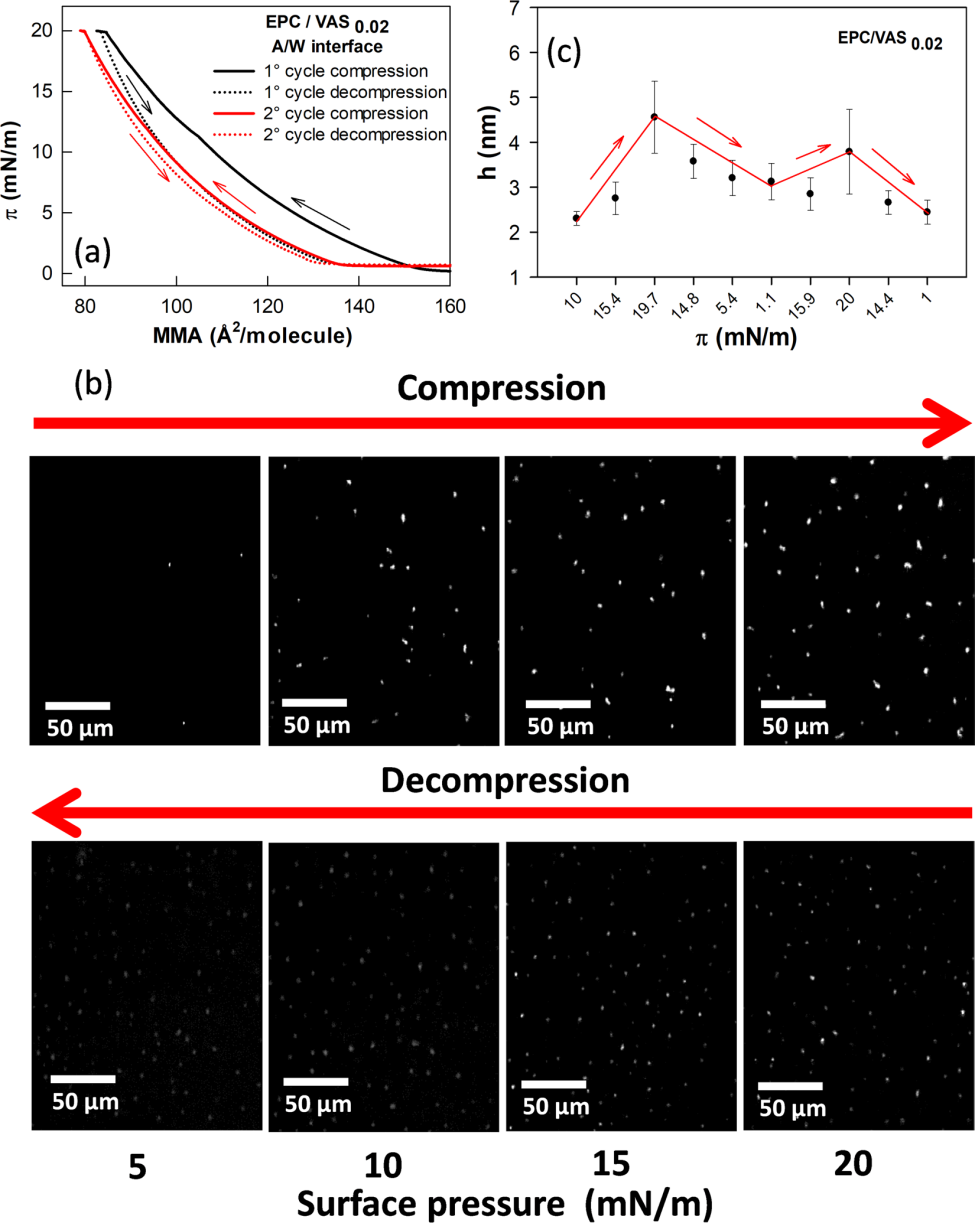
Analysis of compression-decompression cycles of EPC/VAS_0.02_ monolayers at the air-water interface. (**a**) π-MMA isotherms. (**b**) BAM images taken at the indicated π. Arrows indicates the direction of π change. Bar scales represent 50 μm. (**c**) Height (*h*) of lenses over the EPC/VAS film at the A/W interface. Data are the mean ± SEM (n = 5). *h* values were calculated from Eq. 6 according to ref.^25^. At least 5 lenses per BAM image were analyzed. Lines and arrows are just to guide the eye.

In the first C-D cycle the isotherm of EPC/VAS_0.02,A/W_ monolayer exhibited a marked negative hysteresis (Fig. 5a). In the second compression the π-MMA isotherm followed almost the same trajectory as the first decompression, so it appeared displaced towards lower molecular areas compared with the first compression isotherm which suggested that the monolayer had lost some molecules at this stage. The second C-D cycle showed no hysteresis indicating that after the first C-D cycle a stabilization was achieved.

Interestingly, BAM images of EPC/VAS_0.02,A/W_ monolayers (Fig. 5b) exhibited small brilliant dots (hereafter termed lenses) which increased in surface density upon compression and did not disappeared during the decompression (Fig. 5b). The height of lenses (Fig. 5c) was calculated from local reflectivity values according to Eq. 6 using data from BAM images taken at different surface pressures within a C-D cycle. The average values corresponding to several lenses from a same image were plotted against π. Although of micrometer radii, these structures exhibited heights within the nanometer range.

Taken together these observations suggest that during the first compression the film suffers irreversible changes associated to the loss of some molecules, presumably VAS, not recovered after decompression. These molecules are expelled from the monolayer and aggregates over it forming lenses that do not coalesce upon compression.

**Figure 6:**
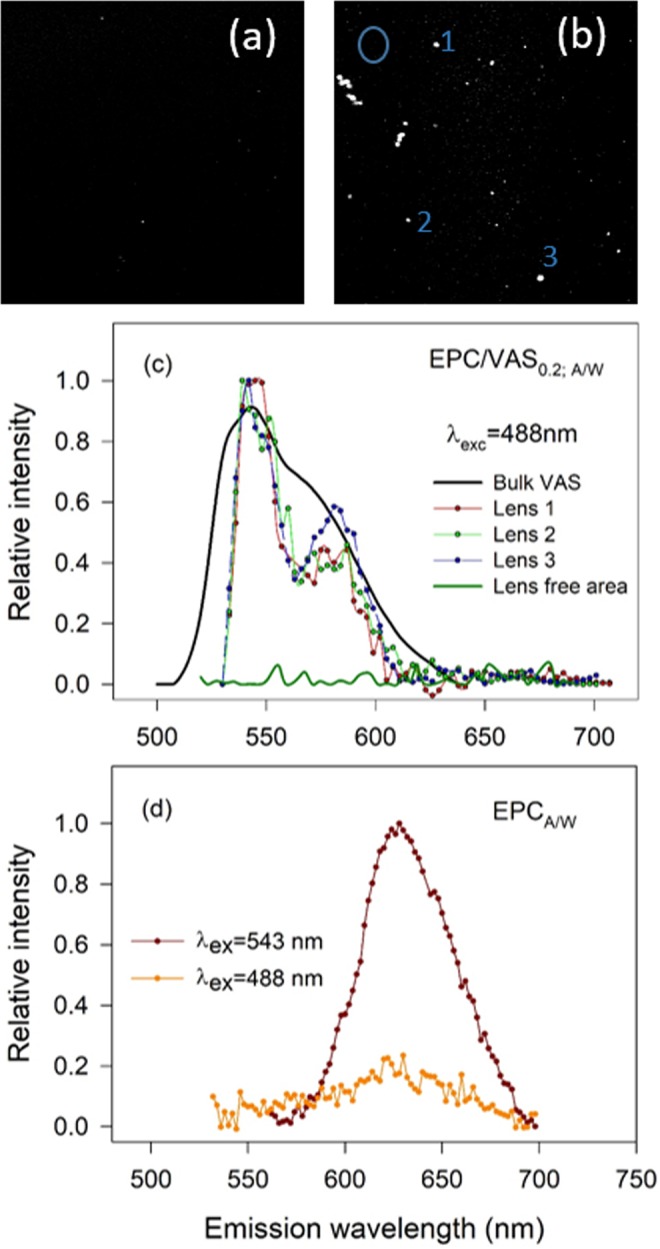
Confocal spectral epifluorescence microscopy of EPC/VAS at the A/W interface. Nile red (NR) was used as fluorescent probe. In all experiments π was ~15–20 mN/m VAS was present in a molar fraction 0.02 (**a**) and 0.2 (**b**). In (**c**) samples emission spectra obtained upon excitation at 488 nm are shown. Spectra were built up from the analysis of the indicated area (*lens* or *lens free area*, indicated in (**b**) by the numbers or the circle, respectively) in successive pictures captured at different λ_em_ within the 500–700 nm range. *Bulk VAS*, Nile red dissolution using VAS as the solvent. (**d**) In pure EPC monolayer Nile red, excited at 488 or 543 nm, exhibited an emission λ_max_ = 628 nm.

To to evaluate if the bright spots identified through BAM corresponded to VAS liquid lenses, we applied Spectral Confocal Microscopy by analyzing monolayers of EPC/VAS mixtures containing Nile-red (NR), a dye known to partition in hydrophobic environments and to exhibit a stong solvatochromism^22,23^. These properties allow evidencing the coexistence of two phases with different polarities (an apolar core of VAS and a less apolar EPC/VAS mixed monolayer). The micrographs (Fig. 6a,b) showed that Nile-red clearly partitioned in the lenses. Note that images of EPC/VAS monolayers with *x*_VAS_ = 0.02 (Fig. 6a) and *x*_VAS_ = 0.2 (Fig. 6b) differed in the bigger size and higher surface density of the lenses observed in the latter compared with the former (coincidently with the patterns found with BAM). The spectral analysis of the dye present in these structures (Fig. 6c), upon excitation at λ_ex_ = 488 nm, demonstrated a fluorescence emission band between 500–670 nm with a maximum (λ_max_), located at ~540 nm which was almost superimposable with that of Nile-red dissolved in bulk VAS. On the contrary, the spectral analysis in regions of EPC/VAS monolayers where lenses were not present (Fig. 6c, dark green line) showed a negligible emission intensity within the 500–670 nm range upon excitation at λ_ex_ = 488 nm. But, when EPC monolayers (Fig. 6d), were excited either at λ_ex_ = 543 nm or at 488 nm (Fig. 6d), we found an emission spectrum band spanned between 565–700 nm with a λ_max_ = 628 nm. This red shifted spectra obtained in EPC monolayers indicated that Nile-red molecules outside the lenses, and within the hydrocarbon chains of EPC, were feeling the effect of an environment more hydrophilic than inside the lenses’ core^22^. It is important to note that the spectra of NR in lenses present in EPC/VAS monolayers containing *x*_VAS_ = 0.02 or 0.2 were qualitatively similar (compare Figs 6c and S2 in the Supplementary Information) but the wider surface of the bigger lenses in the latter improved the signal intensity and thus the spectrum resolution. Taken together these results suggested that NR was distributed along the whole monolayer, inserted between the phospholipid molecules and also inside the lenses. As the emission spectra ressemble that of bulk VAS, we concluded that the lenses were composed of segregated VAS.

After confirming that the highly reflective (thick) structures seen through BAM images are composed of VAS, an analysis based on the spreading over the hydrocarbon chains of the phospholipid monolayer can be done as follows. The spreading coefficient (S) of a liquid over a surface can be consider the difference between the work of adhesion (W_a_) of the liquid to the surface and the work of cohesion (W_c_) of the molecules of the liquid substance among themselves^24,25^. Since W_a_ = *γ*_*AW*_ + *γ*_*AO*_ − *γ*_*OW*_ and W_c_ = 2*γ*_*AO*_ then *S* = *γ*_*AW*_ − (*γ*_*AO*_ + *γ*_*OW*_), where *γ*_*AW*_, *γ*_*AO*_ and *γ*_*OW*_ represent the surface tensions of the air/water, air/VAS and VAS/water interfaces, respectively. The condition for spreading is that S > 0.

In the case of VAS over a monolayer of EPC/VAS_0.02,A/W_ *S* = *γ*_*film*_ − (*γ*_*AO*_ + *γ*_*OW*_). From Eq. 3, at π ~ 15 mN/m the γ_film_ = 57 N/m, γ_AO_ = 33 mN/m and γ_OW_ = 37 mN/m. This gives S = −13 mN/m and predicts that VAS lenses will remain over the EPC/VAS_0.02,A/W_ monolayer and will not spread over it. This was confirmed by the BAM images shown in Fig. 5b. In other words, the formation of VAS lenses implies an energetic cost related to the formation of the three phases in coexistence.

However, if instead of air there is bulk liquid VAS over the monolayer the VAS expelled from the monolayer would be directly integrated into the VAS phase, with a low or no energetic cost. This would explain the lower compression free energy of the EPC_O/W_ monolayer compared with EPC/VAS_0.02,A/W_ which would be related to the difference in the final state of the excess of VAS expelled from the film.

## Conclusions

In the present paper we firstly analyzed three different procedures that had been described in the literature for the preparation of surfactant monolayers at the O/W interface. In two of them the incorporation of the surfactant solution starts after the biphasic solvent system has been prepared and they differ in the interface (A/O or O/W) where the surfactant is deposited. The evolution of the rate constant of π change (*k*) after the addition of each of the successive aliquot of the surfactant solution resulted a useful tool not only to make a rational decision about the best procedure to apply but also provided information about the course followed by the surfactant molecules. Thus, the surfactant adsorption directly on the O/W interface was characterized by an initial regular increase in *k* followed by a decrease and a stabilization at low values that tended asymptotically to zero. This can be envisioned as a clean interface that initially fills rapidly and after reaching a critical molecular density still accepts new molecules but requiring higher activation energy until reaching the “equilibrium pressure”. On the contrary, the procedure consisting on a deposition of the surfactant at the A/O interface exhibited a *k* value fluctuating in a chaotic-like manner. So, π never stabilizes leading to non-reproducible π-MMA isotherms. Empirically, this result was enough to discard this procedure for Langmuir film preparation at the O/W interface. However, the molecular mechanism involved in this interesting non-regular fluctuation in the kinetics of π evolution remains as an opened question. We proposed the hypothesis that this complex behavior emerges from the two dimensions (2D and 3D) involved in the diffusion process associated to this procedure. This would lead to a sort of “hopping” mode mechanism of interfacial diffusion with transition states of the phospholipid molecules transiently detached from the surface. The third procedure applied for film preparation (SM) through the deposition at the A/W interface and a later addition of the upper phase, carefully done, did not introduced irreversible perturbations and leaded to the formation of stable and reproducible films. This fact and the simplicity of its preparation made it the method of choice for the experiments that followed. Contrary to the A/W interface, in the O/W interface highly packed, reproducible monolayers, can be achieved only by compressing a low packed monolayer and not by the successive addition of surfactant molecules.

Then, the formation of EPC films at the O/W interface was performed with the SM method with the aim of yielding compression π-MMA isotherms which were successfully representative of Langmuir monolayers. This allowed the behavior of EPC monolayers to be characterized comparatively in different interfaces. The different energy required to drag EPC_O/W_ and EPC_A/W_ monolayers along the corresponding interface may be reflecting either the differences in the viscosities of the upper phase in each case and/or an extra work required to expel VAS molecules intercalated between EPC hydrocarbon chains in EPC_O/W_ which is absent in EPC_A/W_. So, to shed light on this problem we studied the EPC/VAS_0.02_ mixture at A/W interface. Interestingly, according to calculations from π-MMA, EPC/VAS_0.02_ monolayer exhibited the highest values of compressional energy and the lowest compressibility moduli K of all the films studied. This may be rationalized as the resultant of two opposed forces. The decreasing solubility of VAS in the EPC monolayers upon compression may be the driving force favoring VAS squeezing out from the monolayer phase which is reflected by the high compressibility of the EPC/VAS_0.02_ mixed film. At the same time, the higher the physicochemical similarity between the squeezed molecules and the media where they are bowed in, the lower may be the force and lower the work required for compression. Alternatively, the difference may be explained by the energy required to form nucleation sites in the EPC/VAS_0.02_ mixed film

BAM analysis showed that upon compression VAS segregated over the monolayer, forming non-coalescent strongly reflective lenses that remained after decompression and whose heights’ change (increase/decrease) accompanied the compression/decompression cycle. Taken together, our results indicated that, in EPC_O/W_, VAS molecules from the upper phase inserted between the hydrocarbon chains of the phospholipid. Some VAS molecules remained at the interface up to the collapse but others squeezed out towards the VAS bulk phase with an energy requirement lower than towards the air. VAS squeezed from EPC/VAS_0.02;A/W_ did not spread over the monolayer as predicted by the spreading coefficient S = −13 mN/M and demonstrated by BAM.

Concluding, in the present work we defined rationally the best procedure to prepare phospholipid monolayers at the O/W interface, explained same molecular aspects of the phenomena underneath the experimental methods and provide some knowledge useful to build up experimental models to design and study emulsion particles and lipid droplets. The formers are of interest for drug delivery and the latter the factors that stabilize the different structures related to particles of biochemical, biological and physiological importance such as lipoproteins, leucoplasts and many more, in cells of animal and plant origin.

## Material and Methods

### Materials

L-α-Phosphatidylcholine from Egg, Chicken (EPC) was purchased from Avanti Polar Lipids, Inc. (Alabama, U.S.A) and Nile red (NR) was from Sigma-Aldrich. (All other reagents were of analytical grade (99% pure) and used without further purification. The water was purified by a double-deionization system to yield a product with a conductance of ~0.0 ± 0.1 µS/cm. The light mineral oil (Vaseline, VAS), commercially known as Technical Liquid Petrolatum 70/80 SSU, was from VASEPLUS S.A. (Buenos Aires, Argentina). VAS was composed of saturated alkanes with a chain length between 19 and 29 carbon atoms (25 ± 3 C) and a 347 ± 9 g/mole mean molecular mass as determined by GC-MS, ^1^H-NMR and ^13^C-NMR - DEPT 135° spectra (not shown).

### Methods

#### Langmuir films experiments

Monomolecular layers were prepared and monitored essentially as described previously^4,26^ using a Minitrough II (KSV Instruments Ltd., Finland) equipment. The set up included Teflon^TM^ troughs and Delrin^TM^ barriers that differed in the design depending on the type of film to be studied (at the air/water (A/W) and oil/water (O/W) interface). The barriers moved synchronously by electronic switching. Monolayers were compressed isometrically at a constant low rate of 5 mN.m^−1^.min^−1^ (~4 mm/min.) until reaching the target pressure. A lower compression rate 1 mN.m^−1^.min^−1^ (2 mm/min) was tested, and identical results were obtained. The signal corresponding to the surface area (determined according to the relative position of the two compression barriers) and the output from the surface pressure (π) transducer (a Wilhelmy plate, platinized Pt foil 5 mm wide × 20 mm long × 0.025 mm thick) were measured automatically by the Minitrough and fed into a personal computer through a serial interface using a specific software. Paper made Wilhelmy plates were also used with dissimilar results (not shown). Reproducibility was within ±0.1 nm^2^ and ±1 mN/m for molecular area and π, respectively. Before each experiment the trough was rinsed and wiped with 96% ethanol and several times with bidistilled water. The absence of surface-active compounds in the pure solvents and in the subphase solution (bidistilled water) was checked before each run by reducing the available surface area to less than 10% of its original value after enough time was allowed for the adsorption of possible impurities that might have been present in trace amounts.

#### Monolayers at the air-water (A/W) interface

In experiments with monolayers at the A/W interface, a rectangular trough with 274 cm^2^ total area (36.5 cm length, 7.5 cm wide and 0.75 cm depth) was used, with two moving barriers seated on the upper edge of the trough. Between 5 and 60 µL of a chloroformic solution of lipids was spread over an unbuffered aqueous subphase. About 5 min were allowed for the solvent evaporation before starting the compression.

#### Monolayers at the oil/water (O/W) interface

In the case of studies with monolayers at the O/W interface we used a homemade rectangular trough (231 cm^2^ total area, 31.2 cm length and 7.4 cm width) with a 0.5 cm internal step to seat the two barriers and mark the limit of the lower phase height. The barriers were crossed transversally by tunnels sculpted every 0.5 cm to allow the upper phase flow freely^27^.

It is noteworthy that at the O/W interface the Pt plate suspended from the sensitive microbalance is partially immersed in the water and the oil phases^16^. Hence, to assure stable measurements the plate must be previously wetted in the lower phase solvent.

**Figure 7:**
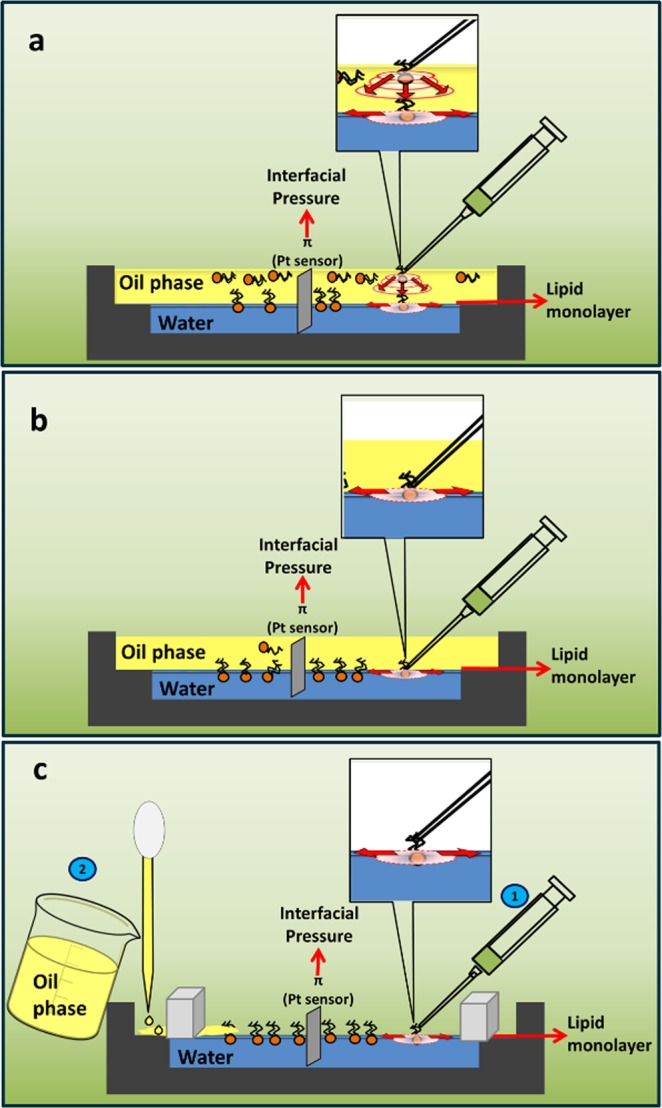
Preparation of adsorbed (AM) (**a**,**b**) and spread (SM) (**c**) monolayers at the O/W interface. The lipid solution was deposited over either the upper phase (**a**) or at the O/W interface after preparing the liquid-liquid biphasic system (**b**) or was spread onto the A/W interface before pouring the oil to obtain the O/W interface (**c**). Insets: (**a**) molecules of phospholipid diffuse radially both in the volume of the oil phase and along the O/W interface; (**b**,**c**) radial diffusion occurs bi-dimensionally along the O/W or the A/W interfaces, respectively.

For the lipid film preparation, three different techniques to build adsorbed (AM) or spread monolayers (SM)^28^ were tested. The AM were formed either by pouring the phospholipid dissolution on the air-oil phase (AM_A/O_) or over the previously formed O/W interface (AM_O/W_) (see Fig. 7a,b). In contrast, to build the SM, the lipid solution was spread at the A/W interface to form a monolayer and after 10 minutes, to allow solvent evaporation, the oil was placed carefully over the water phase with a Pasteur pipette to obtain the O/W interface (Fig. 7c). Note that, in method AM_A/O_, it would be required that the amphipathic surfactant poured over the upper surface have no stability at the A/O interface and to be insoluble in the oil phase to assure that all the deposited molecules reach the O/W interface (Fig. 7a).

### Theoretical calculations

#### Spreading kinetics of phospholipid (PL) at the O/W interface

After the addition of a fixed amount of molecules over the interface, π follows a temporal evolution (π(t)) from an initial value π_0_ at time t = 0 until reaching the equilibrium value π_eq_ or π_max_ when t → ∞ through a maximal increment in π (∆π_max_), as described by Eq. 1:

1 $$\pi (t)={\pi }_{0}+{\rm{\Delta }}{\pi }_{max}\cdot (1-{e}^{-kt})$$

The profile of π(t) will depend on the amount of PL molecules that reach the interface and are sensed by the Wilhelmy plate. So, the profile of π(t) will be affected by the magnitude of the bidimensional diffusion coefficients of PL along the corresponding interface: O/W interface in AM_O/W_ film preparation method and both A/O and O/W interface in the AM_A/O_ method (see Inserts in Fig. 1a,b).

If the preparation method is AM_A/O_ the diffusion rate (*V*_*PL*_) of the PL across de upper oil phase should also be taken into account (Insert in Fig. 1a). *V*_*PL*_ will depend on the 3D diffusion coefficient of PL in oil (*D*_*PL*_), the concentration gradient of PL across the oil phase (∇[*PL*]_*O*_) and the thickness of the oil phase (*h*_*O*_).

2 $${V}_{PL}={D}_{PL}\cdot \frac{\nabla {[PL]}_{O}}{{h}_{O}}$$

For lateral diffusion at interfaces the parameters *D*_*PL*_ in Eq. 2 would refer to the 2D-diffusion coefficient of lipid molecules (*D*_*O/W*,*PL*_) while ∇[*PL*]_*O*_ and *h*_*O*_ should be taken as the gradient of surface molecular density and the distance from the point of spreading to the sensor, respectively.

Information on *D* and *h*_*O*_ (both in the volume and in the surface) are contained in the value of the kinetic constant *k* (Eq. 1) (see Supplementary Information for a further analysis).

#### Mean molecular area (MMA) and surface or interfacial pressure (π)

Mean molecular area (MMA) of phospholipids at different surface pressures was calculated dividing the trough area confined within the barriers by the number of molecules spread on the interface. The surface pressure of an air-water interface (A/W) or the interfacial pressure (π) of an O/W interface (O/W) is defined as follows (Eq. 3):

3 $${\rm{\pi }}={{\rm{\gamma }}}_{0}-{\rm{\gamma }}$$

where γ_0_ and γ are the surface/interfacial tension in the absence and presence of a monolayer, respectively^29^. At 25 °C, γ_0_ is γ_0,A/W_ = 71.9 ± 0.4 mN/m for the clean A/W interface^30^ and γ_0,O/W_ = 37 ± 2 mN/m for the clean O/W interface where O is light mineral oil (VAS, present work).

#### Compressibility modulus

In order to analyze the elastic behavior of the films, the compressibility modulus (*K*) was calculated as follows^31^:

4 $$K=-\,A\,{(\frac{d\pi }{dA})}_{T}$$

where A represents the total monolayer area. For Langmuir isotherms, the dπ/dA data was obtained from regular compression experiments. Note that, in the case of EPC_O/W_ and EPC/VAS_0.02;A/W_, the calculations are made considering an apparent MMA, thus the K will be considered also as an apparent compressibility modulus.

### Thermodynamic analysis

The free energy of compression (*ΔG*_*comp*_) operationally represents the two-dimensional work involved in bringing together the film-forming molecules from a loosely packed state (e.g. π ~ 2 mN/m) to a determined intermolecular packing condition^32^. Then, *ΔG*_*comp*_ can be calculated as:

5 $${\rm{\Delta }}{G}_{comp}=-\,{\int }_{{A}_{0}}^{{A}_{i}}\,\pi \delta A$$

The molecular area (A) can be taken as the MMA and thus *ΔG*_*comp*_ can be calculated as the area under the compression curve between certain limits. In this work, we have taken A_0_ and A_i_ as the MMA at 2 and 20 mN/m, respectively.

### Brewster Angle Microscopy (BAM)

#### Image acquisition

Monolayers were spread over a Langmuir film balance described above and observed during compression and decompression using BAM with an EP3 Imaging Ellipsometer (Accurion, Goettingen, Germany) with a 20X objective (Nikon, NA 0.35). In order to be able to quantify the reflectivity of bright dots, laser shutter and signal gain were adjusted to achieve non-saturated images.

#### Calculation of the height (h) of lenses observed in BAM images of EPC/VAS monolayer at the air-water interface

*h* calculation was based on the procedure described in ref.^33^ from the reflectivity value (*R*_*p*_) of the incident light with λ = 530 nm over a single lens, according to Eq. 6, where η, η_1_ and η_2_ are the lens (1.47), air (1) and subphase (1.33) refractive indexes, respectively, and *θ*_*B*_ is the experimental Brewster angle. At least 5 lenses randomly distributed in each BAM image were measured. Images were taken at different π along two Compression-Decompression cycles within the 1–20 mN/m range.

6 $$h=\frac{\sqrt{{R}_{p}}}{\sin (2{\theta }_{B}-90)}{(\frac{\pi \sqrt{{{\rm{\eta }}}_{1}^{2}+{{\rm{\eta }}}_{2}^{2}}({{\rm{\eta }}}_{1}^{2}-{{\rm{\eta }}}^{2})({{\rm{\eta }}}_{2}^{2}-{{\rm{\eta }}}^{2})}{{({{\rm{\eta }}}_{1}^{2}-{{\rm{\eta }}}_{2}^{2})}^{2}})}^{-1}$$

### Confocal spectral epifluorescence microscopy

Monolayers of EPC/VAS mixtures (at a molar fraction *x*_VAS_ = 0.02 or 0.2) containing 1% NR fluorescent probe were spread over an air /water interface up to a molecular surface density corresponding to a monolayer at ~15–20 mN/m. Then, monolayers were imaged with a spectral confocal laser scanning microscope Olympus FluoView™ FV1000 (Olympus Latin America), equipped with a 10x objective and 488 and 543 nm laser lines, among others. Images were acquired and processed with Olympus FluoView FV10-ASW 3.1 version software, and image editing was carried out using Fiji software (http://fiji.sc/Fiji, Biomedical Imaging Group, EPFL).

## Supplementary information

SUPPLEMENTARY INFORMATION Langmuir films at the oil/water interface revisited

## Data Availability

All data generated or analyzed during this study are included in this published article (and its Supplementary Information Files). Raw data of compression isotherms can be obtained on request to the authors.
