# Supplementary material for: Langmuir films at the oil/water interface revisited
Source: Sci Rep. 2019 Feb 19;9:2259. doi: 10.1038/s41598-019-38674-9 (PMC6381208; doi:10.1038/s41598-019-38674-9)
Supplement: Supplementary file 1 — SUPPLEMENTARY INFORMATION Langmuir films at the oil/water interface revisited [file 41598_2019_38674_MOESM1_ESM.pdf]

SUPPLEMENTARY INFORMATION

Langmuir films at the oil/water interface revisited

*Milagro Mottola, Benjamín Caruso and Maria A. Perillo**

[a] Universidad Nacional de Córdoba. Facultad de Ciencias Exactas, Físicas y Naturales. Depto. de Química, Cátedra de Química Biológica. Córdoba, Argentina. Av. Vélez Sársfield 1611, X5016GCA Córdoba, Argentina

[b] CONICET, Instituto de Investigaciones Biológicas y Tecnológicas (IIBYT). Córdoba, Argentina.

*Corresponding author: mperillo@unc.edu.ar

CONTENTS (S1 to S3 refer to page numbers)

S1 - Title, authors, affiliations and description of supporting information content.

S2 - Diffusion at the O/W interface

S3 - Normalized surface pressure-mean molecular area compression.

S4 - Confocal spectral epifluorescence microscopy of EPC/VAS at the A/W interface.

Diffusion at the O/W interface

From the Einstein relation, $D = k_B T/\lambda$. For the case of a 2D-diffusion between two liquid phases it can be defined a $D_{O/W,PL}$ which has been related to the viscosities of both bulk phases η_{water} (1 cP) and η_{oil} (1.9 cP for VAS)[1] through a power law defined by Eq.3 [2]:

$$D_{O/W,PL} \sim (\eta_{water} + \eta_{oil})^{-0.85} \qquad [3]$$

Also, a modified hydrodynamic drag term $\lambda = 8(\eta_1 + \eta_2)R$ for the Einstein relation has previously been experimentally verified to be an accurate model for lipid objects with radius R in a lipid monolayer between two viscous media with viscosities η_1 and η_2 [3].

The complexity of the phenomenon of lipid diffusion at the liquid-liquid interface resides in the fact that $\Delta[PL]_O$ and R change continuously upon compression as well as upon adsorption, depending on the experiment.

in cP $\quad$ $D \sim 1.7258.10^{-11}.T/[(\eta 1 + \eta 2)R]$ m^2/s $\quad$ or $\quad$ $D \sim 0.17258.T/[(\eta 1 + \eta 2)R]$ µm^2/s

For η_{water} = 1 cP and η_{VAS} = 1.9 cP (r.f.[16]), T=294K and R=3.99, it results that $D = 4.38.10^{-10}$ m^2/s 438 µm^2/s. This value compares reasonably with data from [2].

References

1. http://www.engineersedge.com/fluid_flow/fluid_date.thm.
2. Negishi, M.; Seto, H.; Hase, M.; Yoshikawa, K., How Does the Mobility of Phospholipid Molecules at a Water/Oil Interface Reflect the Viscosity of the Surrounding Oil? *Langmuir* **2008,** *24*, 8431-8434.
3. Walder, R. B.; Honciuc, A.; Schwartz, D. K., Phospholipid Diffusion at the Oil−Water Interface. *Journal of Physical Chemistry B* **2010,** *114* (35), 11484–11488.

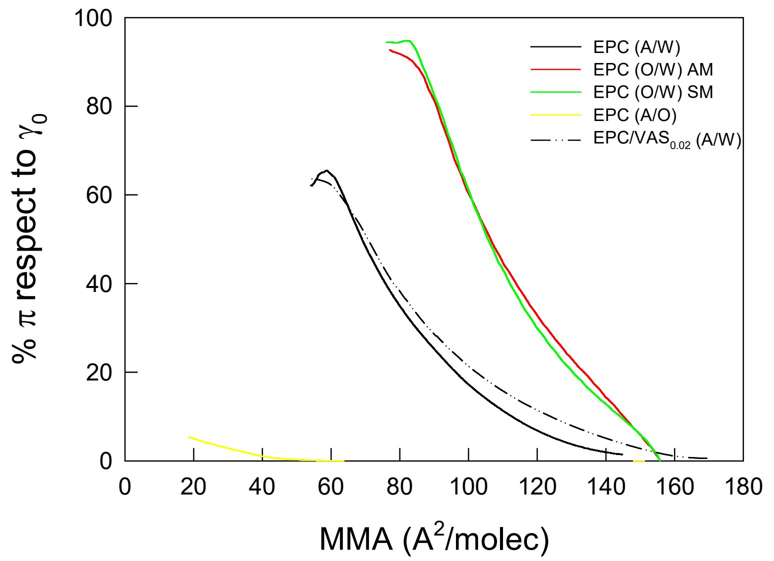

Figure S1. Surface pressure-mean molecular area compression isotherms of EPC at the A/W, A/O and O/W interfaces and EPC/VAS$_{0.02}$ mixture at the A/W interface, normalized by the surface tension of the corresponding free interface (γ_0).

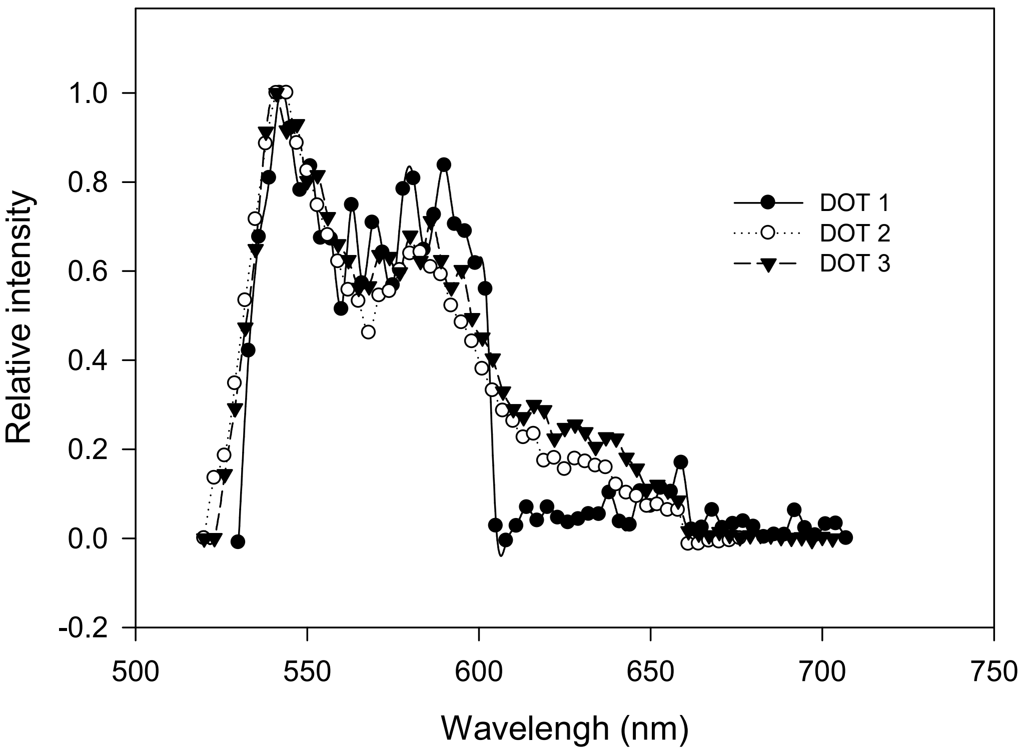

Figure S2. Confocal spectral epifluorescence microscopy of EPC/$VAS_{0.02}$ at the A/W interface Nile red was used a fluorescent probe. VAS was present in a molar fraction 0.2.

Samples emission spectra obtained upon excitation at 488 nm are shown. Spectra were built up from the analysis of three different lenses in successive pictures captured at different λ_{em} within the 500-700 nm range.
